# H3ABioNet computational metagenomics workshop in Mauritius: training to analyse microbial diversity for Africa

**DOI:** 10.1186/s40793-015-0111-0

**Published:** 2015-12-01

**Authors:** Shakuntala Baichoo, Gerrit Botha, Yasmina Jaufeerally-Fakim, Zahra Mungloo-Dilmohamud, Daniel Lundin, Nicola Mulder, Vasilis J. Promponas, Christos A. Ouzounis

**Affiliations:** H3ABioNet & H3Africa Consortium, University of Mauritius, Reduit, 80837 Mauritius; Department of Integrative Biomedical Sciences, Computational Biology Group, IDM, H3ABioNet & H3Africa Consortium, University of Cape Town Faculty of Health Sciences, Anzio Road, Observatory, 7925 Cape Town, South Africa; Centre for Ecology and Evolution in Microbial model Systems, Linnaeus University, 39182 Kalmar, Sweden; Department of Biological Sciences, Bioinformatics Research Laboratory, New Campus, University of Cyprus, PO Box 20537, CY-1678 Nicosia, Cyprus; Biological Computation & Process Laboratory (BCPL), Chemical Process Research Institute (CPERI), Centre for Research & Technology (CERTH), PO Box 361, GR-57001 Thessalonica, Greece

## Abstract

In the context of recent international initiatives to bolster genomics research for Africa, and more specifically to develop bioinformatics expertise and networks across the continent, a workshop on computational metagenomics was organized during the end of 2014 at the University of Mauritius. The workshop offered background on various aspects of computational biology, including databases and algorithms, sequence analysis fundamentals, metagenomics concepts and tools, practical exercises, journal club activities and research seminars. We have discovered a strong interest in metagenomics research across Africa, to advance practical applications both for human health and the environment. We have also realized the great potential to develop genomics and bioinformatics through collaborative efforts across the continent, and the need for further reinforcing the untapped human potential and exploring the natural resources for stronger engagement of local scientific communities, with a view to contributing towards the improvement of human health and well-being for the citizens of Africa.

## Background

The Human Heredity and Health in Africa [1] is a major international initiative whose primary objectives are (i) to facilitate genomics research on genetic and infectious disease for African populations engaging with the local communities and expertise and (ii) to contribute towards capacity building for the entire African continent [2]. There have already been some important results for human genomics research, addressing various aspects of human diversity, historical population shifts, and medical genomics [3]. Other projects involve health research [4], infectious disease [5] and bioinformatics training [6]. The latter is supported by the H3ABioNet project [7].

H3ABioNet seeks to establish a pan-African bioinformatics network to both develop the field of computational biology through the sharing of infrastructures and training, as well as to support ongoing research projects of the H3Africa consortium [6]. The H3ABioNet network, which is part of the H3Africa consortium, currently consists of 32 Bioinformatics research groups distributed amongst 15 African countries and 2 partner institutions based in the USA.

With regard to bioinformatics training, H3ABioNet organizes a number of workshops relevant to H3Africa research. These have covered a diverse range of topics from biological data management and bioinformatics core services to human genetic variation and genomics [8]. In this context, we proposed to organize and obtained support for a computational metagenomics workshop at the University of Mauritius towards the end of 2014.

Prior to the CMW in 2014, the University of Mauritius coordinated the implementation of a capacity building program in bioinformatics, which was funded by SANBio. From 2010 to 2012, several training workshops were held in Mauritius and South Africa, with the collaboration of the University of Pretoria and University of Cape Town, South Africa, the University of Uppsala, Sweden and CST-IT, Finland. The main themes of these training activities were on Pathogen Genomics, Phylogenetics, Next Generation Sequencing and Genome Assembly and Annotation. Participants were university academics and researchers from eleven countries in southern Africa. This initiative resulted in an increased awareness for including bioinformatics in university curricula as well as motivating research students in engaging in projects on genomics.

The latest follow-up on advanced training, was a workshop on metagenomics with international speakers which took place recently. The Computational Metagenomics Workshop was held from 1–5 December 2014 at the University of Mauritius at Reduit-Mauritius, providing a week-long series of training seminars, practical demonstrations, hands-on tutorials and research talks on the use of software tools to analyze and interpret metagenomics data.

## Purpose and program

The main aim of the workshop was to provide participants with cutting-edge fundamental theory and practical applications of metagenomics. We envisaged a comprehensive program to ensure that all participants would have a certain degree of knowledge regarding operating systems, hardware/software basics, algorithms and key principles of bioinformatics and computational biology, before advancing towards the data resources, tools and applications of metagenomics for health and the environment.

The full program was announced on the H3ABioNet website [9].

## Instructors and content

A programme was conceived by Prof. Christos Ouzounis (CERTH, Greece) and further adapted in consultation with all coauthors of this report, and significant contributions from instructors Shakuntala Baichoo (University of Mauritius), Gerrit Botha (University of Cape Town, South Africa), Daniel Lundin (Linnaeus University, Sweden) and Vasilis Promponas (University of Cyprus, Nicosia, Cyprus). The programme covered topics on Computational Metagenomics, namely: fundamentals of biology, computing and bioinformatics; introduction to Linux; principles of molecular structure, alignment and sequence comparison; metagenomics essentials, platforms and data analysis; and finally thematic research areas in metagenomics.

As mentioned above, we aimed at a thorough introduction to the basics of bioinformatics before proceeding into the more advanced aspects of computational metagenomics. The latter included an introduction to the conceptual aspects of genome, metagenome and pangenome analysis, new sequencing technologies, sequence annotation and pathway inference, the description of various community initiatives worldwide, and practical demonstration of tools for metagenomics data analysis and interpretation.

## Announcement and applications

The workshop was advertised on the University of Mauritius website [10], and the websites of H3ABioNet (as above) and SANBio [11]. It was also circulated by email to specific mailing lists. By the deadline 43 applications were received from various African countries. There were 17 applications from SADC countries [12], including Mauritius and 26 from other African countries, which are members of H3ABioNet or H3Africa. Applications arrived from 11 countries in Africa, namely: Botswana, Egypt, Ethiopia, Kenya, Malawi, Mauritius, Morrocco, Sudan, South Africa, Uganda, and Zimbabwe.

## Applicants and selection

A committee from the University of Mauritius evaluated the SADC and Mauritian applications based on the research directions of candidates and selected 5 candidates from the SADC (excluding South Africa) and 5 local applicants from Mauritius.

The remaining 26 applications were sent to H3ABioNet where the selection of H3ABioNet and H3Africa candidates was made and a further 10 applications were deemed to be most appropriate. Hence a total of 20 participants were chosen for the workshop.

## Activities and analysis

The workshop was held over five days, from 9 am to 6 pm. On the first four days, the morning periods were dedicated to imparting theoretical knowledge, while the afternoon periods were devoted to hands-on practical sessions.

On Monday, an overview of the fundamentals of genome biology and Linux programming were provided. This was to provide the essential background information required for participants to follow the course. We covered the fundamentals of biological sciences, computing, and computing with biological data. The practical introduced Linux and a pre-compiled image was shared for hands-on work and the practicals that followed.

On Tuesday, we covered fundamentals of bioinformatics and practical applications, based on a well-known bioinformatics textbook. This material was based on contributions from two instructors/co-authors (VJP, CAO) for a previous EMBO workshop, also listed on H3ABioNet [13], held in Athens during May 2014 [14]. The practical illustrated aspects of sequence comparison and analysis based on prepared examples.

On Wednesday, metagenomics was introduced in conjunction with genome and pangenome analysis, as well as software platforms for metagenomics data interpretation, which were covered in greater detail. The practical session illustrated the use of widely used software tools for alignment and taxonomic categorization.

On Thursday, major metagenomics platforms being developed elsewhere were presented. The practical focused on 16S analysis, alignment and classification. The participants now had the chance to put everything into practice, and discuss both opportunities and challenges associated with metagenomics research with their own projects at their local institutions.

On Friday, the last day of the workshop, case studies were further discussed from ongoing research at the instructors’ laboratories. There were also specific presentations on thematic areas of metagenomics and four presentations from participants on their research projects. Additionally, the participants were organised in small groups of 3 to 4 and were asked to study select papers that interested them and later present them to the entire class, as a journal club activity. We encouraged extensive interaction between the participants, with the hope that further collaborative research projects will emerge from this workshop. The final outcome of the more loosely structured part of the course was a focus on pairwise collaboration between the participants’ laboratories and an informal network of colleagues, with a newly established emailing list.

**Fig. 1:**
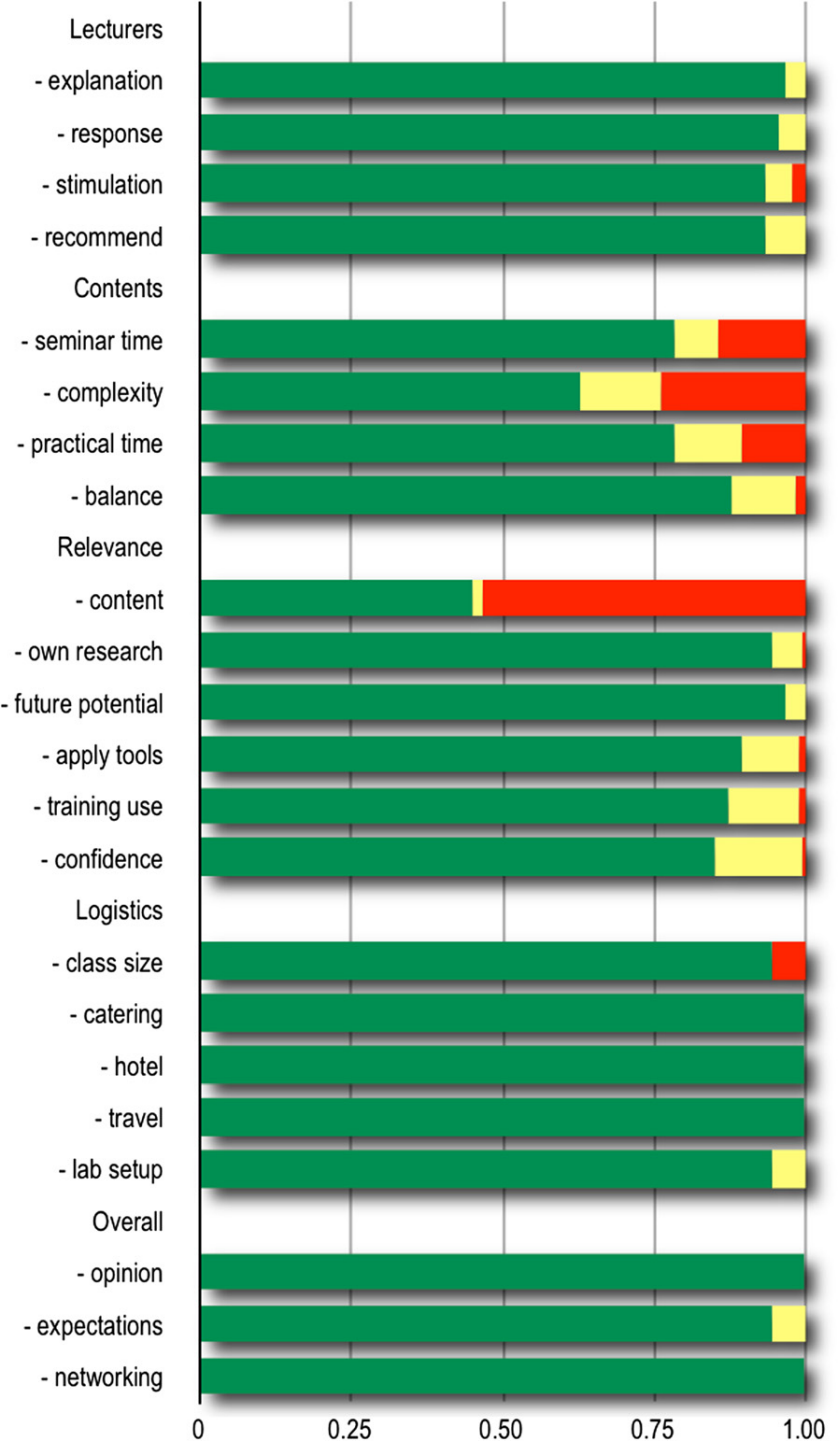
A graphical representation of the questionnaire feedback statistics. Responses were broadly codified on a relative scale (poor-red, satisfactory-yellow, excellent-green) from 0 to 1. Most participants provided very positive views about the lecturers, content and logistics. The relatively low grades for the relevance (specifically, content) category are due to the extensive introductory material that was presented, to ensure that everyone achieved a similar level of understanding to follow the course successfully. Based on feedback stastitics, future versions of the workshop contents can be adapted to participant needs and backgrounds

In order to measure the level of knowledge assimilation, pre- and post-workshop questionnaires with technical questions were circulated to the participants. A feedback system was also setup to collect the views of participants (Fig. 1). We discovered that the knowledge of the participants was quite substantial in various aspects of bioinformatics, as the extensive introductory material provided was quite familiar to most. We also realized that most applicants, being biologists, were less familiar with command-line work and programming, an issue that will need to be tackled in future events of a similar nature.

## Conclusions and recommendations

Overall, the event, which was branded as CMW, was very successful, as judged by the participant responses. The local organization was excellent and no major logistical obstacles were encountered (Fig. 1). Participants appreciated the traditional hospitality, the natural beauty and the pleasant weather of Mauritius as well as the good training infrastructure and the commitment of the University to run such a successful course. We have understood better the needs of the participants, who came from prestigious institutions across Africa, and their project requirements. The untapped human potential of the continent must be further developed, with contributions from the H3Africa consortium and other collaborating institutions. We discovered that many participants had an unexpected level of deeper understanding of advanced computational biology principles and that future events of a similar nature might choose to focus more on the practical side rather than introductory material.

In conclusion, the workshop contributed towards the facilitation of metagenomics research by engaging researchers from laboratories across the continent providing expertise and relevant content, and hopefully provided additional capacity building in the form of collaborative efforts between the participants, thus fulfilling the two primary goals of the H3Africa initiative. We feel that the CMW workshop can form the basis for a series of training workshops where the material can be further adapted and perhaps advanced, as the local expertise in the field increases. Finally, we have made an effort to compile most of the material into an online resource accessible via the Web, with most contributions made publicly available (to the extent possible), including—importantly—the Ubuntu/QIIME VirtualBox image with data and software already pre-packaged for further use by the wider community. The site is accessible at the H3ABioNet site [15]. Further feedback is most welcome by direct email to the corresponding authors of this report.

## Abbreviations

H3Africa: Human Heredity & Health in Africa, an initiative to build capacity for genomics research in Africa
H3ABioNet: Pan African Bioinformatics network for H3Africa
CMW: Computational Metagenomics Workshop
SANBio: Southern Africa Network for Biosciences
SADC: Southern African Development Community
